# Artificial intelligence in ophthalmology: from innovation to clinical integration

**DOI:** 10.3389/fopht.2026.1839194

**Published:** 2026-04-30

**Authors:** Bharat Gurnani, Kirandeep Kaur

**Affiliations:** 1 Department of Cataract, Cornea, Ocular Surface and Refractive Surgery, Neemuch, Madhya Pradesh, India; 2 Department of Cataract, Pediatric Ophthalmology, Strabismus and Neuro-Ophthalmology, Neemuch, Madhya Pradesh, India

**Keywords:** artificial intelligence, deep learning, multimodal imaging, ophthalmology, precision medicine

## Abstract

Artificial intelligence (AI) has emerged as a transformative force in modern ophthalmology, enabling rapid advances in disease detection, clinical decision support, workflow optimization, and tele-ophthalmology. Ophthalmology is particularly suited for AI integration because of its reliance on imaging modalities such as fundus photography, optical coherence tomography (OCT), and visual field testing. Over the past decade, deep learning algorithms have demonstrated high diagnostic accuracy in identifying retinal diseases including diabetic retinopathy, age-related macular degeneration, and glaucoma. The approval of autonomous AI diagnostic systems for diabetic retinopathy screening marked a significant milestone in clinical adoption. Beyond diagnostics, AI is increasingly influencing surgical planning, predictive analytics, education, and patient engagement. Despite these promising advances, significant challenges remain regarding algorithm generalizability, ethical considerations, regulatory approval, data privacy, and integration into routine clinical practice. This perspective article reviews current innovations in AI applications within ophthalmology and discusses their clinical impact while outlining future directions for research and implementation. We argue that the next phase of AI in ophthalmology will involve multimodal learning systems, integration with large language models, and deployment in global eye-care networks to address disparities in access to care. A collaborative approach involving clinicians, data scientists, regulators, and industry will be essential to ensure safe, ethical, and effective adoption of AI technologies in ophthalmic practice.

## Introduction

Artificial intelligence (AI) has rapidly evolved from an experimental computational concept to a powerful clinical tool across multiple medical specialties (1). Among these fields, ophthalmology has emerged as one of the most promising domains for AI applications (2). This is largely due to the specialty’s dependence on digital imaging modalities, standardized diagnostic criteria, and the availability of large annotated datasets (2). Machine learning and deep learning algorithms can analyze high-resolution ocular images and identify patterns that may not be easily detectable by clinicians (3). The global burden of visual impairment continues to rise, driven by aging populations, increasing prevalence of diabetes, and limited access to eye-care services in many regions. Early detection and timely intervention remain critical in preventing avoidable blindness (4). AI has the potential to transform screening programs and expand access to eye care by enabling automated disease detection and remote diagnostic support. One of the most significant milestones in AI adoption in ophthalmology was the regulatory approval of autonomous AI systems capable of diagnosing diabetic retinopathy from retinal images (5). The system IDx-DR (now LumineticsCore) became the first autonomous AI medical device approved by the U.S. Food and Drug Administration in 2018, demonstrating high diagnostic performance in detecting referable diabetic retinopathy.

Subsequently, several additional AI-based screening tools such as EyeArt and AEYE-DS have received regulatory clearance, further validating the clinical feasibility of AI-assisted ophthalmic diagnostics (6). Beyond retinal diseases, AI algorithms are increasingly applied in glaucoma detection, anterior segment imaging, refractive surgery planning, and surgical training. Advances in multimodal AI models integrating data from OCT, fundus photography, and clinical parameters are further expanding the potential of AI-driven ophthalmic care. To contextualize the clinical impact of artificial intelligence in ophthalmology, it is important to consider quantitative benchmarks of regulatory approval and real-world deployment. As of recent reports, ophthalmology ranks among the leading medical specialties in terms of FDA-cleared AI-based devices, largely driven by its image-based diagnostic framework (7). Multiple autonomous and assistive AI systems for diabetic retinopathy screening including IDx-DR (LumineticsCore), EyeArt, and AEYE-DS have received regulatory clearance, with reported sensitivities and specificities approaching or exceeding 85–90% in real-world settings. In comparison to other specialties such as radiology and cardiology, ophthalmology demonstrates a unique advantage due to the availability of standardized imaging modalities and well-defined disease endpoints, facilitating faster translation from algorithm development to clinical deployment (8). However, despite a growing number of approvals, the proportion of AI systems achieving widespread routine clinical integration remains relatively limited across all medical domains. This highlights a critical gap between regulatory validation and real-world implementation, emphasizing the need for robust post-deployment evaluation, workflow integration, and health system adaptation. The rapid clinical translation of artificial intelligence in medicine is reflected in the growing number of regulatory approvals. Recent analyses indicate that over 900–950 AI/ML-enabled medical devices have received FDA authorization, with a marked exponential increase since 2016 (9). Notably, radiology accounts for approximately 70–76% of all approved AI devices (over 700 devices), making it the most dominant specialty in this domain. In contrast, ophthalmology represents a relatively smaller but highly impactful segment, with early regulatory breakthroughs such as autonomous diabetic retinopathy screening systems establishing it as one of the first specialties to achieve real-world deployment of AI-driven diagnostics. Despite the numerical dominance of radiology, ophthalmology has demonstrated a disproportionately high level of clinical integration and autonomy, owing to standardized imaging modalities and well-defined disease endpoints. This contrast highlights a key distinction between volume of approvals and depth of clinical implementation, emphasizing that regulatory clearance does not necessarily equate to widespread adoption or clinical utility (6). In this perspective article, we explore the evolving role of artificial intelligence in ophthalmology, highlighting recent technological innovations, clinical impact, and emerging opportunities. We also discuss the challenges that must be addressed to ensure responsible and effective integration of AI into routine ophthalmic practice (10).

## Current advances in artificial intelligence in ophthalmology

Artificial intelligence advancements in ophthalmology can be broadly categorized into technology-driven developments and clinical application-based implementations. Technology-oriented advances include the evolution of deep learning architectures, multimodal imaging systems, explainable artificial intelligence, and data infrastructure frameworks that form the backbone of modern AI systems (2, 3). In parallel, clinical applications span across multiple ophthalmic subspecialties, including anterior segment disorders, retinal diseases, glaucoma, pediatric ophthalmology, neuro-ophthalmology, refractive surgery, and surgical workflow optimization. For clarity and conceptual continuity, the following section is structured to first highlight major clinical applications, followed by emerging technological paradigms that are shaping the future of AI integration in ophthalmology (5, 6).

## Clinical applications of artificial intelligence in ophthalmology

### Artificial intelligence in anterior segment and ocular surface disorders

While much of the early success of artificial intelligence in ophthalmology has been driven by retinal imaging, increasing attention is now being directed toward the anterior segment, where AI is beginning to demonstrate significant diagnostic and predictive capabilities (10). High-resolution slit-lamp photography, anterior segment OCT, and corneal topography provide rich datasets that can be leveraged for machine learning applications. In corneal diseases such as keratoconus, AI algorithms have shown remarkable ability to detect subclinical and forme fruste disease, which is often missed by conventional diagnostic criteria (11). Advanced models trained on tomographic and biomechanical parameters can identify early ectatic changes, enabling timely intervention and reducing the risk of postoperative complications in refractive surgery. Similarly, AI has been applied to classify infectious keratitis based on slit-lamp images, differentiating bacterial, fungal, and viral etiologies with promising accuracy (12). This has particular relevance in regions where access to microbiological diagnostics is limited. In ocular surface disorders, AI-based systems are being developed to quantify tear film instability, meibomian gland dysfunction, and ocular surface staining patterns. Automated analysis of meibography images allows objective grading of gland dropout, which may improve the diagnosis and monitoring of dry eye disease (13). Furthermore, AI-driven platforms may enable remote assessment of ocular surface conditions through smartphone-based imaging, expanding access to care in underserved settings. The integration of AI into anterior segment diagnostics is expected to enhance clinical decision-making, standardize grading systems, and facilitate early detection of disease, thereby improving patient outcomes (14).

### Artificial intelligence based retinal disease screening

Retinal diseases have been at the forefront of AI research in ophthalmology. Diabetic retinopathy (DR), age-related macular degeneration (AMD), and retinal vascular disorders are particularly well suited for automated analysis due to their characteristic imaging features. Deep learning algorithms trained on large datasets of fundus photographs have achieved diagnostic accuracy comparable to human graders in detecting diabetic retinopathy (5, 15, 16). Autonomous AI systems can analyze retinal images in real time and provide referral recommendations without human oversight. These technologies have demonstrated sensitivities and specificities approaching 90% in detecting referable diabetic retinopathy, highlighting their potential as reliable screening tools (17). The clinical impact of such systems is particularly significant in primary care and community screening settings. AI-assisted screening can facilitate early disease detection, reduce specialist workload, and improve referral efficiency. Studies have also demonstrated that AI-driven clinical decision support systems can enhance referral accuracy and reduce unnecessary consultations (18). In low-resource environments, AI-enabled screening platforms integrated with portable fundus cameras or smartphone imaging systems have the potential to expand access to eye care (19). Emerging programs have already demonstrated high detection accuracy in community-based screening initiatives (19, 20). Most clinically validated systems in retinal imaging are based on convolutional neural networks trained on large annotated datasets, with architectures specifically optimized for fundus image classification. These models are typically narrow in scope but demonstrate high performance within defined tasks, reinforcing the continued relevance of task-specific deep learning in real-world clinical deployment. These regulatory milestones underscore the transition of AI from experimental validation to clinically deployable tools, positioning ophthalmology as one of the earliest specialties to achieve real-world implementation of autonomous AI systems (21).

### Artificial intelligence in glaucoma and optic nerve disease

Glaucoma remains one of the leading causes of irreversible blindness worldwide. Early detection is challenging because structural and functional damage may occur before clinical symptoms appear (2). AI has shown promise in analyzing optic nerve head images, retinal nerve fiber layer thickness measurements, and visual field data to aid in early diagnosis and disease monitoring (22, 23). Deep learning algorithms can detect subtle structural changes in the optic nerve and predict glaucoma progression using OCT and fundus images (20, 22). AI-based models have demonstrated strong potential in identifying early glaucomatous changes and stratifying patients based on risk profiles. Recent research also suggests that AI could transform glaucoma management by enabling predictive analytics that estimate disease progression and treatment response (24). Such approaches may allow clinicians to tailor management strategies according to individual patient risk profiles, supporting a shift toward precision medicine (25). In glaucoma, machine learning models have been widely applied to structured datasets such as visual fields and OCT-derived parameters, where algorithms such as random forests and support vector machines have demonstrated strong predictive capabilities for disease progression and risk stratification (21).

### Artificial intelligence in pediatric ophthalmology and amblyopia

Pediatric ophthalmology presents unique challenges, including limited patient cooperation, variability in examination techniques, and the critical importance of early detection. Artificial intelligence offers a powerful tool to address these challenges by enabling objective, rapid, and non-invasive screening (26). AI-based vision screening tools utilizing smartphone cameras and portable imaging devices can detect amblyogenic risk factors such as refractive error, strabismus, and media opacities (3). These systems are particularly valuable in community and school-based screening programs, where access to trained personnel may be limited. Early identification of amblyopia is crucial, as timely intervention during the critical period of visual development can significantly improve outcomes (27). Recent advances also include AI models capable of analyzing eye alignment and ocular motility patterns from video recordings, providing quantitative assessments of strabismus. Such tools may reduce interobserver variability and improve the accuracy of diagnosis (28). In addition, AI has potential applications in monitoring treatment adherence in amblyopia therapy. Digital platforms equipped with machine learning algorithms can track patching compliance and provide real-time feedback to caregivers, enhancing treatment effectiveness (29).

### Artificial intelligence in neuro-ophthalmology

Neuro-ophthalmology represents a complex intersection between ophthalmology and neurology, where diagnosis often relies on subtle clinical signs and multimodal investigations. AI has the potential to assist clinicians in this domain by integrating imaging, functional testing, and clinical data (2). Asaoka et al. described that machine learning algorithms have been applied to analyze visual field patterns, identifying characteristic defects associated with optic neuropathies, chiasmal lesions, and cortical visual disorders (23). Medeiros et al. showed that AI-driven analysis of OCT data can detect thinning of the retinal nerve fiber layer and ganglion cell complex, aiding in the diagnosis of optic nerve diseases (22). As per Milea et al, AI may assist in differentiating papilledema from pseudopapilledema using fundus imaging, a clinically challenging distinction with significant implications for patient management. Integration of neuroimaging data with ophthalmic findings may further enhance diagnostic accuracy (30).

### Artificial intelligence in refractive surgery and biometry

Artificial intelligence is poised to revolutionize refractive surgery by improving the accuracy of preoperative assessment and postoperative outcomes (2). Traditional intraocular lens (IOL) power calculation formulas are based on regression models that may not fully account for individual anatomical variability (31). AI-based biometry models, trained on large datasets, can incorporate multiple parameters to generate more accurate predictions (32). In refractive surgery, machine learning algorithms can analyze corneal topography, wavefront data, and biomechanical properties to optimize surgical planning (33). AI-driven systems may recommend personalized ablation profiles, reducing the risk of postoperative refractive surprises and enhancing visual outcomes. Additionally, predictive analytics can identify patients at higher risk of complications such as ectasia, enabling more informed patient selection and counseling (34).

### Emerging technologies and foundational artificial intelligence frameworks

While the aforementioned sections highlight subspecialty-specific clinical applications, recent advances in artificial intelligence are increasingly driven by underlying technological innovations. These include multimodal learning systems, foundation models, explainable AI frameworks, and integrated data ecosystems, which are collectively redefining the scope and scalability of AI in ophthalmology (1, 7, 10).

### Multimodal imaging and foundation models

**Figure 1:**
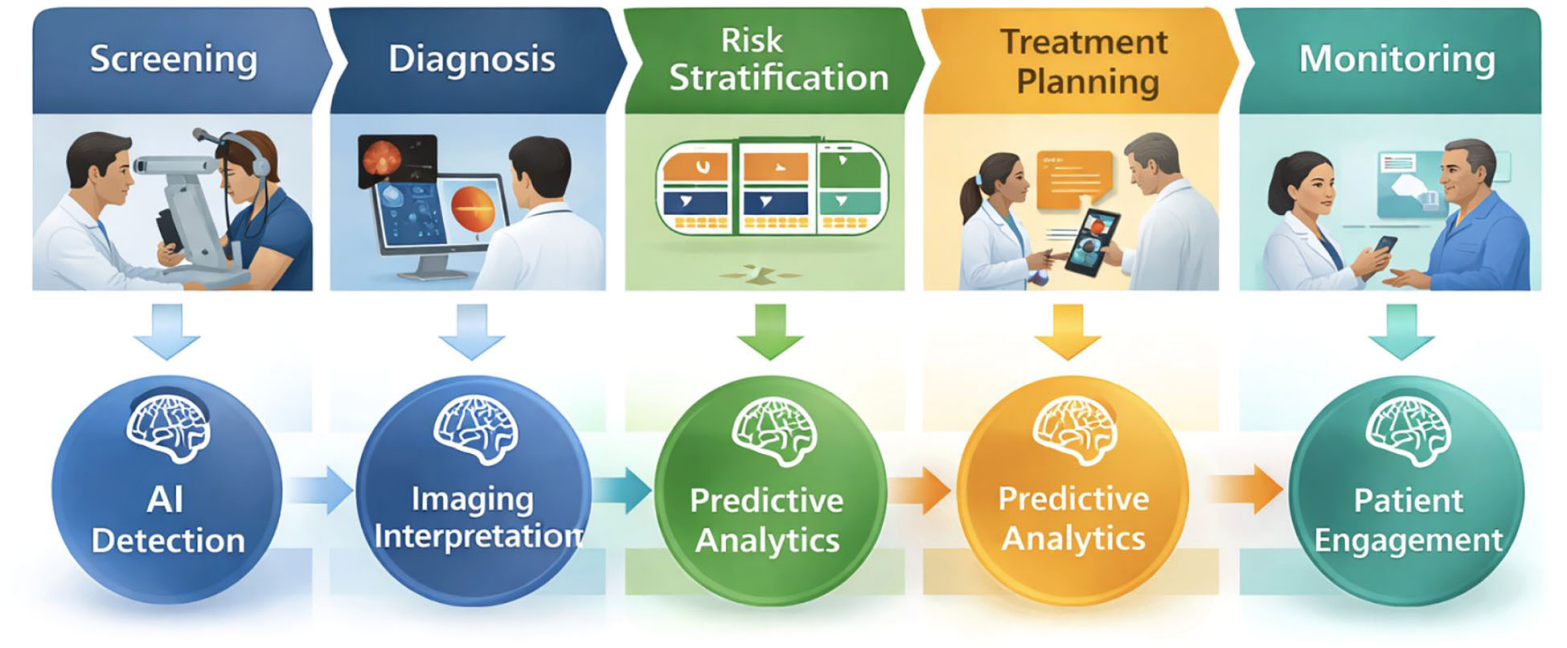
Artificial intelligence ecosystem in ophthalmology. The colored schematic diagram illustrates integration of multimodal data with deep learning to enable clinical decision support and precision care.

While recent discourse in artificial intelligence has increasingly focused on foundation models and large-scale multimodal architectures, it is important to recognize that the current clinical landscape in ophthalmology is predominantly driven by task-specific deep learning and machine learning models. Convolutional neural networks (CNNs) remain the backbone of most deployed systems, particularly in retinal disease screening, where models are optimized for classification tasks such as diabetic retinopathy detection, age-related macular degeneration grading, and referable disease identification (2, 3, 15, 16). In parallel, traditional machine learning approaches including support vector machines, random forests, and gradient boosting techniques continue to play a significant role in analyzing structured clinical data such as visual field indices, intraocular pressure trends, and biometric parameters. This distinction underscores that, while foundation models represent an important future direction, current clinical impact is largely driven by highly optimized, domain-specific AI systems (10, 22, 24). One of the most exciting developments in AI research is the emergence of multimodal learning systems capable of analyzing diverse ophthalmic datasets simultaneously. These systems integrate multiple imaging modalities including OCT, OCT angiography, and fundus photography along with clinical data to improve diagnostic performance (35). Recent multimodal foundation models trained on millions of ophthalmic images have demonstrated the ability to perform diverse tasks including disease classification, visual question answering, and risk prediction (36). These models learn generalizable representations of ocular pathology and may eventually support comprehensive automated ophthalmic assessment. Such advances represent a shift from task-specific algorithms toward more versatile AI systems capable of supporting multiple clinical functions within a single framework (1) (**Figure 1**). Despite their versatility, foundation models are still in the early stages of clinical translation, with challenges related to interpretability, computational requirements, and real-world validation. In contrast, task-specific models continue to dominate clinical practice due to their robustness, efficiency, and ease of integration into existing workflows (8).

### Artificial intelligence in ophthalmic surgery and clinical workflow

**Figure 2:**
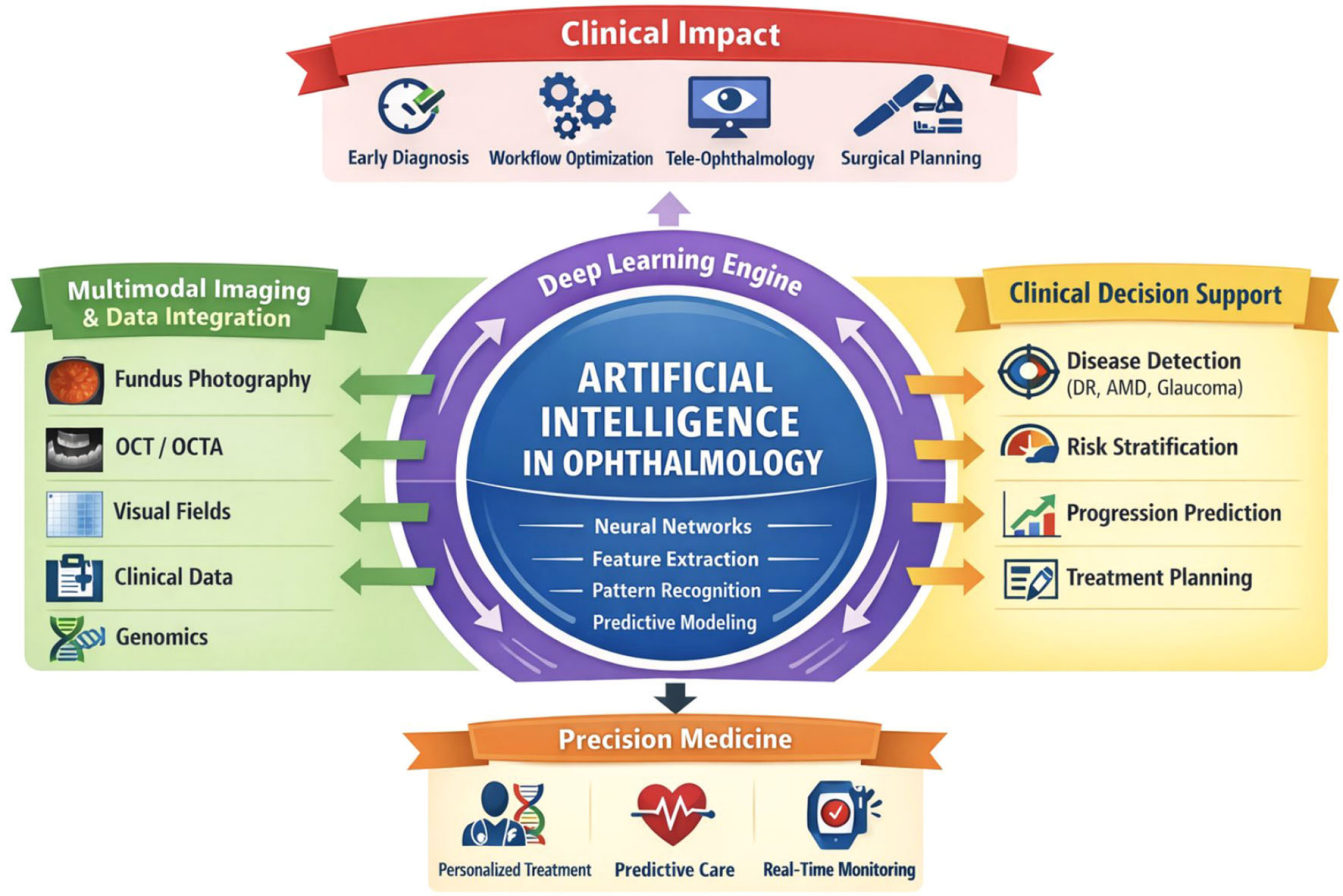
Artificial intelligence integration across the ophthalmic care continuum. The colored schematic diagram illustrates the role of AI from screening to monitoring, enabling automated detection, predictive analytics, treatment planning, and enhanced patient engagement.

Artificial intelligence is increasingly influencing ophthalmic surgical practice and clinical workflow management. AI-driven systems can assist surgeons in preoperative planning, intraoperative guidance, and postoperative monitoring (37). In cataract and refractive surgery, machine learning algorithms have been applied to predict surgical outcomes and optimize intraocular lens (IOL) power calculations (32). AI-based biometric formulas are demonstrating improved accuracy in predicting postoperative refractive outcomes compared with traditional regression models. AI also has potential applications in surgical video analysis (38). By analyzing surgical recordings, machine learning systems can identify procedural steps, assess surgical skill, and provide feedback for training purposes. These technologies may play a significant role in ophthalmic education and simulation training (38). Furthermore, AI-based clinical decision support systems can streamline clinical workflows by prioritizing urgent cases, triaging referrals, and assisting clinicians in interpreting imaging results (**Figure 2**). These application-driven advances are increasingly supported and enhanced by next-generation AI architectures, particularly multimodal and foundation models, which enable integration across diverse datasets and clinical workflows (15).

### Explainable artificial intelligence and trust in clinical practice

As AI systems become more integrated into clinical workflows, the need for explainability and transparency becomes increasingly important. Clinicians must understand how AI models arrive at their decisions to trust and effectively utilize these tools (39). Explainable AI techniques, such as heat maps and feature attribution methods, provide insights into the regions of an image that influence model predictions. These tools can help validate AI outputs and identify potential sources of error. Building trust in AI systems will require not only technical advances but also robust validation studies, clear communication of limitations, and ongoing clinician engagement (40).

### Artificial intelligence in ophthalmic education and research

Artificial intelligence is beginning to reshape ophthalmic education and research (1, 2). AI-driven platforms can assist trainees in learning diagnostic interpretation, analyzing imaging datasets, and developing research insights. Recent studies have highlighted the role of AI-assisted educational tools in enhancing ophthalmology training programs by providing automated feedback and adaptive learning systems (2). Large language models and specialized ophthalmology-focused AI assistants are also emerging as tools to support clinical decision-making and patient communication. These systems may assist clinicians in summarizing clinical data, generating reports, and answering patient queries. While these technologies remain in early stages, they hold promise for enhancing medical education and supporting evidence-based clinical practice (10).

### Artificial intelligence and health equity

One of the most significant opportunities for AI in ophthalmology lies in its potential to address disparities in access to eye care (4, 14). However, this potential can only be realized if AI systems are developed and deployed in an equitable manner. Bias in training datasets can lead to disparities in algorithm performance across different populations (7, 18). Ensuring diversity in datasets and conducting rigorous external validation are essential to mitigate this risk. AI-enabled tele-ophthalmology platforms can expand access to screening and diagnostic services in underserved regions (14, 17). However, infrastructure limitations, such as internet connectivity and device availability, must be addressed to ensure equitable implementation (4).

### Human artificial intelligence collaboration: the hybrid model

The future of ophthalmology is likely to be defined by human–AI collaboration, where clinicians and AI systems work together to achieve optimal outcomes (1, 2). Rather than replacing clinicians, AI will augment their capabilities by providing rapid analysis, reducing cognitive load, and supporting decision-making (1). Hybrid models that combine AI outputs with clinician expertise have been shown to outperform either approach alone (18). This synergy highlights the importance of integrating AI into clinical workflows in a manner that enhances, rather than disrupts, patient care (7).

### Data infrastructure and interoperability

The success of AI in ophthalmology will depend on the development of robust data infrastructure and interoperability standards (2, 10). Integration of AI systems with electronic health records, imaging devices, and clinical databases is essential for seamless workflow integration (10, 18). Standardization of data formats and imaging protocols will facilitate data sharing and improve model performance (10). Collaborative data-sharing initiatives may accelerate AI development while ensuring patient privacy and data security (1, 18).

### Regulatory evolution and lifecycle monitoring

Regulatory frameworks for AI-based medical devices are evolving to address the unique challenges posed by adaptive algorithms (1, 2). Traditional approval processes designed for static devices may not be sufficient for systems that continuously learn and update (2). Future regulatory approaches are likely to emphasize lifecycle monitoring, real-world performance evaluation, and post-market surveillance (18). Establishing clear guidelines for validation and updating of AI systems will be critical to ensure safety and effectiveness (10, 18).

### Economic implications and value-based care

Artificial intelligence has the potential to transform the economics of ophthalmic care by improving efficiency and reducing costs (1, 2). Automated screening and triage systems can reduce the burden on specialists and optimize resource utilization (5, 6). However, the implementation of AI technologies requires significant investment in infrastructure, training, and maintenance (11). Cost-effectiveness analyses will be essential to evaluate the long-term value of AI in clinical practice (18). Integration of AI into value-based care models may further enhance its adoption by aligning financial incentives with improved patient outcomes (1, 10) (**Table 1**).

**Table 1:** Depicts the major AI applications in ophthalmology: current status and future potential.

S. No	Domain	Current applications	Key limitations	Future potential
1	Retina	DR, AMD screening, OCT analysis	Generalizability, device variability	Real-time monitoring, predictive therapy
2	Glaucoma	RNFL analysis, visual field prediction	Early disease detection variability	Progression modeling, risk stratification
3	Cornea	Keratoconus detection, infectious keratitis classification	Limited datasets	Personalized refractive planning
4	Pediatric ophthalmology	Amblyopia screening, strabismus detection	Compliance, data scarcity	Home-based monitoring systems
5	Neuro-ophthalmology	Visual field interpretation, optic neuropathy detection	Complex multimodal integration	Integrated neuro-visual diagnostics
6	Surgery	IOL calculations, surgical video analysis	Limited intraoperative adoption	AI-guided robotic surgery
7	Tele-ophthalmology	Remote screening platforms	Infrastructure limitations	Global AI-driven care networks

## Discussion

Artificial intelligence represents one of the most significant technological advancements in the history of ophthalmology (1, 2). The field has already witnessed important milestones, including the regulatory approval of autonomous diagnostic systems and the integration of AI into screening programs and clinical decision support tools (5, 15, 16). However, AI should not be viewed as a replacement for ophthalmologists but rather as a complementary technology that augments clinical expertise (1). Human oversight remains essential for interpreting AI outputs, managing complex cases, and ensuring patient-centered care (10, 18). From a clinical perspective, the most impactful applications of AI may lie in population-level screening and early disease detection (4, 15, 16). By enabling scalable and cost-effective screening programs, AI technologies could play a pivotal role in reducing the global burden of preventable blindness (4). At the same time, the responsible integration of AI into ophthalmology will require careful attention to ethical considerations, algorithm transparency, and equitable access to technology (7, 18). Collaborative efforts between clinicians, engineers, policymakers, and industry partners will be essential to ensure that AI innovations translate into meaningful improvements in patient outcomes (1, 2). A critical consideration in current AI development is the distinction between emerging foundation models and established task-specific algorithms. While foundation models offer the promise of generalizable intelligence, the majority of clinically validated and deployed systems in ophthalmology continue to rely on task-specific architectures, reflecting the importance of targeted performance and clinical reliability (14). In conclusion, artificial intelligence is poised to reshape the landscape of ophthalmic practice. As technological capabilities continue to evolve, the integration of AI into routine clinical care has the potential to enhance diagnostic accuracy, improve healthcare efficiency, and expand access to eye care worldwide (2, 17). The challenge ahead lies not only in developing sophisticated algorithms but also in ensuring their safe, ethical, and equitable implementation in clinical practice (1, 15) (**Table 2**). Interestingly, while radiology dominates in terms of absolute number of FDA-approved AI systems, ophthalmology remains one of the earliest specialties to achieve clinically autonomous AI deployment, highlighting a unique balance between regulatory approval and real-world clinical translation (5, 8). Notably, ophthalmology has emerged as one of the first specialties to achieve regulatory approval of fully autonomous AI diagnostic systems, reflecting its suitability for image-based deep learning applications and standardized clinical endpoints (5, 15, 16). **Table 3** enlist the regulatory landscape of AI-Enabled medical devices across specialties.

**Table 2:** From hype to reality: critical appraisal of AI in ophthalmology.

S. No	Domain	Current perception	Ground reality	Key gap	Future direction
1	Diagnostic accuracy	Near-human or superior performance	High accuracy in controlled datasets	Reduced performance in real-world settings	Robust external validation across populations
2	Screening programs	AI can replace human graders	Works best as triage tool, not replacement	Workflow integration challenges	Hybrid AI–clinician screening models
3	Glaucoma detection	Early detection breakthrough	Moderate performance in early disease	Structural-functional discordance	Longitudinal progression modeling
4	Surgical AI	AI will automate surgery	Limited to planning and analytics	Lack of real-time intraoperative AI	AI-guided robotic microsurgery
5	Tele-ophthalmology	Universal access solution	Dependent on infrastructure	Connectivity and device limitations	Offline-capable and edge AI systems
6	Multimodal AI	Comprehensive disease understanding	Early-stage integration	Data standardization issues	Unified multimodal clinical platforms
7	Explainability	AI decisions are interpretable	Most models still “black box”	Low clinician trust	Explainable AI (XAI) integration
8	Regulation	AI approvals increasing rapidly	Static approvals dominate	Adaptive AI not fully regulated	Lifecycle-based regulatory frameworks
9	Cost-effectiveness	AI reduces healthcare costs	High initial investment required	Maintenance and scaling costs	Value-based reimbursement models
10	Global impact	AI will reduce blindness worldwide	Uneven adoption across regions	Health inequity and data bias	Inclusive datasets and global deployment

**Table 3:** Depicts the regulatory landscape of AI-Enabled medical devices across specialties.

S. No	Parameter	Ophthalmology	Radiology	Other specialties
1	Approximate FDA-approved AI devices	~9–20 devices	>700 devices	~200 devices combined
2	Proportion of total AI devices	<5%	~70–76%	~20–25%
3	Dominant application	Fundus imaging (DR, AMD)	CT, MRI, X-ray analysis	Cardiology, neurology, pathology
4	Level of autonomy	High (autonomous DR screening systems)	Mostly assistive	Mostly assistive
5	Clinical integration	Moderate to high (screening programs)	Variable	Limited
6	Data standardization	High (structured imaging)	High (multi-modality imaging)	Variable
7	Key limitation	Limited diversity of indications	Limited clinical validation	Infrastructure + validation gaps

## Challenges and limitations

Despite the transformative potential of artificial intelligence in ophthalmology, several critical challenges continue to hinder its seamless clinical adoption (2, 10). One of the foremost concerns is data quality and generalizability, as AI models trained on limited or homogeneous datasets may fail to perform reliably across diverse populations, imaging devices, and real-world clinical settings, underscoring the need for robust external validation (10, 18). In parallel, ethical and regulatory considerations including issues of transparency, accountability, and patient data privacy remain complex, with regulatory frameworks struggling to keep pace with rapid technological advancements (1, 40, 41). Equally important is clinical integration, where the success of AI depends on its ability to align with existing workflows and electronic health systems, while also ensuring that clinicians are adequately trained to interpret outputs and recognize algorithmic limitations (2, 14). Finally, economic and accessibility barriers persist, as the costs associated with infrastructure, maintenance, and continuous algorithm updates may limit widespread implementation, particularly in resource-constrained settings (4, 14). Together, these challenges highlight that while AI holds immense promise, its true impact will depend on thoughtful, ethical, and clinically grounded integration into ophthalmic practice (1, 10).

## Future directions

The next decade is poised to witness a transformative expansion in artificial intelligence–driven ophthalmology, redefining both diagnostics and therapeutic paradigms (1, 2). Emerging multimodal AI systems will integrate imaging data with clinical and genomic inputs, enabling deeper disease phenotyping and highly personalized management strategies (7, 31). The advent of real-time clinical decision support systems is expected to enhance point-of-care efficiency, offering instantaneous, evidence-based insights during patient consultations (2, 15). Concurrently, AI-powered tele-ophthalmology platforms will play a pivotal role in bridging disparities in global eye care by facilitating automated screening and remote specialist access, particularly in underserved regions (11, 14). The integration of ophthalmology-specific large language models may further streamline clinical workflows by assisting in documentation, literature synthesis, and patient communication (1). Ultimately, these advances will accelerate the shift toward precision ophthalmology, where predictive analytics and individualized therapeutic planning replace conventional population-based approaches, ushering in a new era of intelligent, data-driven, and patient-centric eye care (7, 30).

## Expert opinion

Artificial intelligence in ophthalmology has transitioned from proof-of-concept algorithms to clinically deployable systems; however, its real-world impact remains uneven. While diagnostic accuracy has reached near-human performance in controlled settings, the true challenge lies in clinical translation, scalability, and integration into decision-making pathways. In our view, the current emphasis on diagnostic algorithms may represent only the first phase of AI evolution. The next major breakthrough will likely emerge from predictive and longitudinal AI systems that can forecast disease progression and guide individualized treatment strategies. Importantly, AI is at risk of being overvalued in isolated diagnostic tasks while being underutilized in workflow optimization, patient monitoring, and health system integration. The greatest global benefit of AI may not lie in tertiary care centers but in primary care and underserved regions, where automated screening and tele-ophthalmology can dramatically improve access to eye care. Another critical consideration is the gap between algorithm performance and clinical trust. Even highly accurate models will fail to gain adoption unless they are interpretable, reliable across diverse populations, and seamlessly integrated into clinical workflows. Furthermore, regulatory frameworks must evolve to accommodate adaptive AI systems that continuously learn from new data. Looking ahead, the convergence of multimodal AI, wearable diagnostics, and large language models will redefine ophthalmic practice. Rather than replacing clinicians, AI will serve as an intelligent co-pilot, augmenting clinical judgment and enabling more precise, data-driven care. Ultimately, the success of AI in ophthalmology will depend not only on technological innovation but also on ethical implementation, clinician engagement, and equitable global deployment.

## Conclusion

Artificial intelligence is no longer a futuristic concept in ophthalmology—it is an evolving clinical reality with the potential to redefine how eye care is delivered across the globe. From anterior segment diagnostics and pediatric screening to surgical planning and longitudinal disease monitoring, AI is expanding its footprint across every subspecialty. The next phase of innovation will be characterized by integration, personalization, and collaboration, where AI systems operate seamlessly within clinical ecosystems and complement human expertise. However, the successful translation of AI from research to routine practice will require careful attention to data quality, ethical considerations, regulatory frameworks, and clinician training. A multidisciplinary approach involving ophthalmologists, engineers, policymakers, and industry stakeholders will be essential to navigate these challenges. Ultimately, the goal of AI in ophthalmology is not merely to enhance diagnostic accuracy but to improve patient outcomes, expand access to care, and reduce the global burden of visual impairment. As the field continues to evolve, the integration of intelligent technologies into clinical practice holds the promise of a more precise, efficient, and equitable future for eye care.

## Data Availability

The original contributions presented in the study are included in the article/supplementary material. Further inquiries can be directed to the corresponding author.
